# Homeobox proteins are potential biomarkers and therapeutic targets in gastric cancer: a systematic review and meta-analysis

**DOI:** 10.1186/s12885-020-07346-7

**Published:** 2020-09-09

**Authors:** Xiao Jin, Lu Dai, Yilan Ma, Jiayan Wang, Haihao Yan, Ye Jin, Xiaojuan Zhu, Zheng Liu

**Affiliations:** Institute of Digestive Endoscopy and Medical Centre for Digestive Disease, The Second Affiliated Hospital, Nanjing Medical University, Nanjing, Jiangsu Province 210011 People’s Republic of China

**Keywords:** Homeobox proteins, Gastric cancer, Prognosis, Clinicopathological characteristics, Meta-analysis

## Abstract

**Background:**

An increasing number of studies have described the aberrant expression of homeobox (HOX) proteins in gastric cancer (GC), which is critically associated with the prognosis and clinicopathological characteristics of GC. This study was conducted to investigate the clinical value and action mechanisms of HOX proteins in GC.

**Methods:**

A comprehensive search of PubMed, Embase, Web of Science and Cochrane Library was performed in accordance with the Preferred Reporting Items for Systematic Reviews and Meta-Analysis (PRISMA) statement. The pooled hazard ratio (HR) with its 95% confidence interval (95% CI) and the pooled odds ratio (OR) with its 95% CI were used to assess the effect of HOX protein expression on the prognosis and clinicopathological features of GC, respectively.

**Results:**

Nineteen studies containing 3775 patients were selected for this study. Heterogeneity among HRs of overall survival (OS) was markedly high (I^2^ = 90.5%, *p* = 0.000). According to the subgroup analysis, increased expression of HOX protein in the downregulated subgroup was associated with a good prognosis for patients with GC (pooled HR: 0.46, 95% CI: 0.36–0.59, I^2^ = 3.1%, *p* = 0.377), while overexpression of HOX protein in the upregulated subgroup was correlated with a reduced OS (pooled HR: 2.59, 95% CI: 1.79–3.74, I^2^ = 73.5%, *p* = 0.000). The aberrant expression of HOX protein was crucially related to the TNM stage, depth of tumour invasion, tumour size, lymph node metastasis, distant metastasis, vascular invasion, histological differentiation and Lauren classification in patients with GC. In addition, the molecular mechanisms by which HOX proteins regulate tumorigenesis and development of GC were also explored.

**Conclusions:**

HOX proteins play vital roles in GC progression, which might serve as prognostic markers and therapeutic targets for GC.

## Background

Gastric cancer (GC) is one of the most common cancers. Although the incidence of GC is decreasing, it remains the sixth most common malignancy and accounts for 8.2% of global cancer-related deaths, according to the most recent global cancer statistics reported in 2018 [1]. GC is highly heterogeneous, and patients with GC are usually diagnosed in an advanced stage. Despite significant developments in surgical techniques and adjuvant therapy, the overall prognosis of patients with GC is still poor. Therefore, it is urgent to identify molecular markers to predict the prognosis and evaluate the therapeutic effect of GC.

Homeobox (HOX) genes encode a highly conserved family of 39 transcription factors that are divided into four clusters (HOXA, HOXB, HOXC, and HOXD). HOX proteins play crucial roles in the early development of embryos and organs, including vertebrae, external genitalia, gastrointestinal tract and central nervous system [2]. Because embryogenesis and tumorigenesis share similar characteristics such as growth and differentiation, the deregulation of HOX protein has been observed in abnormal development and malignancy [3]. HOX proteins function as oncogenes or tumour suppressors, depending on the context [4]. An increasing number of HOX proteins have been investigated in various tumours, including acute myeloid leukaemia [5], breast cancer [6], lung cancer [7], and digestive tract neoplasms [8–10]. Currently, the implications of HOX proteins in tumorigenesis and development of GC have been reported in many studies. Nevertheless, the roles of HOX proteins in GC remain controversial.

Therefore, considering the conflicting conclusions of current researches, we conducted this systematic review and meta-analysis to explore the prognostic and clinicopathological value of HOX proteins in GC and summarized the molecular mechanisms by which HOX proteins regulate tumorigenesis and development of GC.

## Methods

This study was conducted according to the PRISMA guidelines [11].

### Search strategies

Two researchers (XJ and LD) independently performed a comprehensive literature search of PubMed, Embase, Web of Science and Cochrane Library through March 6, 2020. The following MeSH terms and text words were used in combination: “genes, homeobox” or “gene, homeobox” or “homeobox gene” or “homeobox genes” or “genes, homeotic” or “gene, homeotic” or “homeotic gene” or “hox genes” or “gene, hox” or “genes, hox” or “hox gene” or “genes, homeo box” or “gene, homeo box” or “homeo box gene” or “homeo box genes” or “homeotic genes” or “homeo box sequence” or “homeo box sequences” or “sequences, homeo box” or “homeo boxes” or “sequence, homeo box” or “homeobox sequence” or “homeobox sequences” or “sequence, homeobox” or “sequences, homeobox” or “homeoboxes” or “homeo box” or “homeobox” or “hoxa1” or “hoxa5” or “hoxa9” or “hoxa10” or “hoxa11” or “hoxa13” or “hoxb5” or “hoxb7” or “hoxb8” or “hoxb9” or “hoxb13” or “hoxc6” or “hoxc9” or “hoxc10” or “hoxd4” or “hoxd9” or “hoxd10” or “hoxd13” or “stomach neoplasms” or “neoplasm, stomach” or “stomach neoplasm” or “neoplasms, stomach” or “gastric neoplasms” or “gastric neoplasm” or “neoplasm, gastric” or “neoplasms, gastric” or “cancer of stomach” or “stomach cancers” or “gastric cancer” or “cancer, gastric” or “cancers, gastric” or “gastric cancers” or “stomach cancer” or “cancer, stomach” or “cancers, stomach” or “cancer of the stomach” or “gastric cancer, familial diffuse”. The references of eligible studies in this field were also searched manually. Two investigators (XJ and LD) reviewed the titles and abstracts of studies and retrieved studies that met the inclusion criteria for full-text evaluation.

### Selection criteria

All authors jointly deliberated and set the selection criteria. The following inclusion criteria were established: (1) GC was pathologically and histologically confirmed; (2) HOX protein expression was detected using immunohistochemical (IHC) staining in GC tissues and paired noncancerous mucosae; (3) studies evaluated the correlation between HOX protein expression and the outcome of GC; and (4) studies supplied sufficient information for calculating the HR with its 95% CI for survival and the OR with its 95% CI for clinicopathological parameters. The following exclusion criteria were used: (1) overlapping or duplicate data; (2) reviews, letters, case reports, conference abstracts, retracted articles, editorials, and full texts not published in English; (3) studies of cancer cells or animal models, or irrelevant studies; and (4) studies without adequate information for calculating HRs and 95% CIs.

### Quality assessment

Two researchers (YM and JW) assessed the quality of studies using the Newcastle-Ottawa Quality Assessment Scale (NOS) [12]. The NOS consists of three aspects of evaluation: selection, comparability and outcomes between the case group and the control group. Studies that scored ≥6 points were considered high quality. Any disagreement was resolved by discussion.

### Data extraction

Two investigators (XJ and LD) independently reviewed all included studies and extracted the available data. The following information was collected: (1) publication details, including the first author’s name, publication year and origin of the studied population; (2) characteristics of the studied population, including HRs with 95% CIs and clinicopathological features. In the studies that reported HRs from both univariate and multivariate models, we extracted the HR from the latter model that had been adjusted for potential confounders. If the HR was not reported, it was extrapolated using Engauge Digitizer v.11.1 software, Tierney’s spreadsheet [13], and (3) a cut-off value.

### Statistical analysis

HR and its 95% CI from each study were used to calculate the pooled HR and its 95% CI. The heterogeneity of the combined HR was determined using Cochran’s Q test and Higgins’ I-squared statistics. A *p* value< 0.05 was considered significant. We used the random effects model if heterogeneity was observed (I^2^ ≥ 50%). The fixed effects model was used in the absence of heterogeneity (I^2^<50%) [14]. A subgroup analysis was conducted based on the expression level of HOX proteins in patients with GC. The sensitivity analysis was conducted to evaluate the stability of the results, after excluding each study. Publication bias was assessed using the Begg’s funnel plots and Egger’s tests, and a *p* value< 0.05 was considered significant. Statistical analyses were performed using Stata statistical software (version 15.0).

## Results

### Literature search

**Fig. 1:**
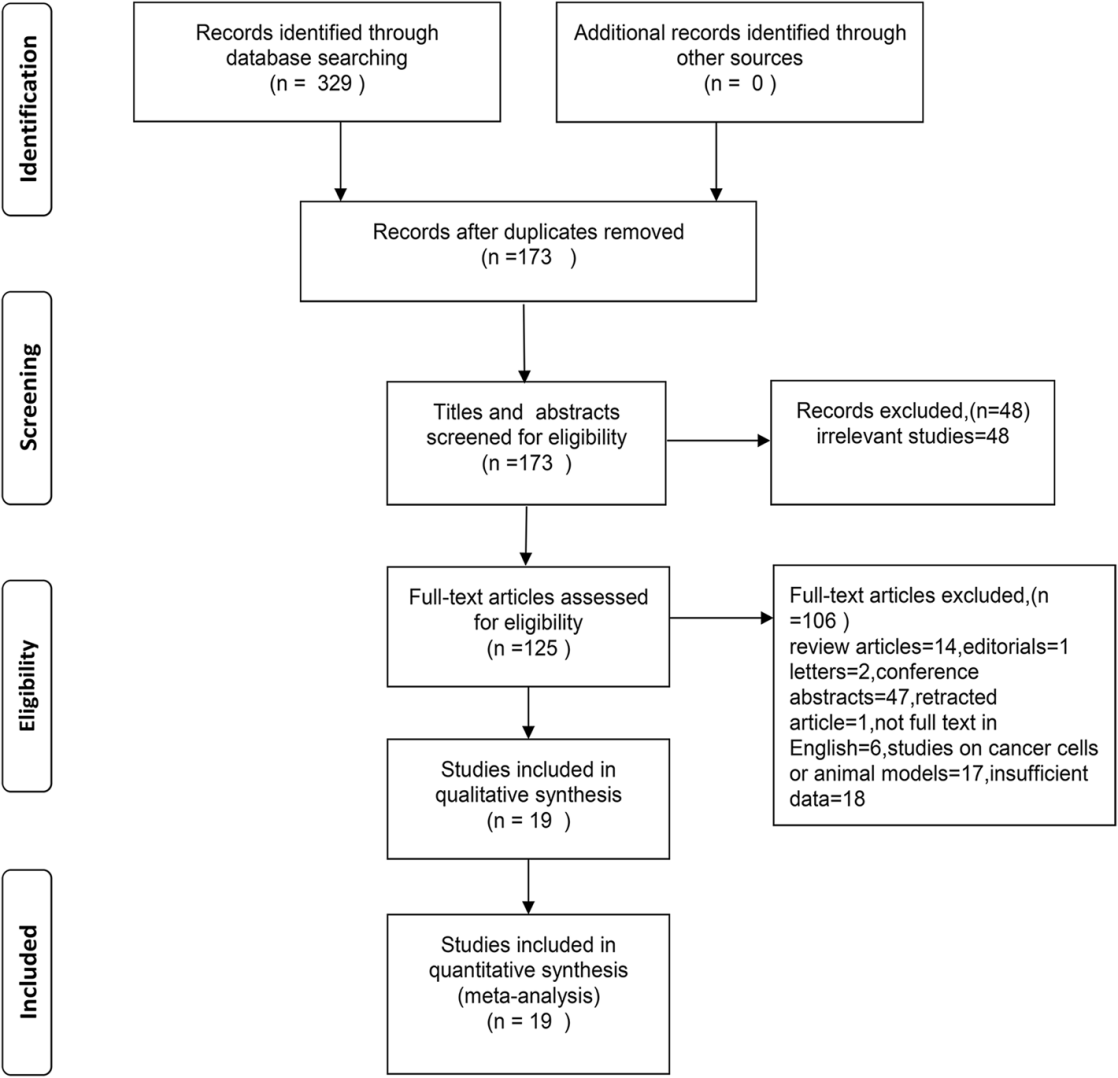
Flow diagram of this systematic review and meta-analysis

Our search strategy preliminarily identified 329 potential records. One hundred seventy-three articles remained after the removal of duplicated studies. Forty-eight of these studies were removed after perusing the titles and abstracts. Then, reviews, editorials, letters, conference abstracts, retracted articles, full texts not published in English, and studies of cancer cells or animal models were excluded. Subsequently, 18 studies lacking insufficient data were rejected. Finally, 19 studies including 3775 patients with GC were included in this analysis. The selection process is shown in Fig. 1.

### Study characteristics

All included studies were conducted in China, Japan and Korea and were published between 2012 and 2019. These studies involve the following HOX proteins: HOXB9 [15], HOXD10 [16], HOXA5 [17, 18], HOXA10 [19–21], HOXA13 [22, 23], HOXC6 [24], HOXB7 [25, 26], HOXA1 [27], HOXA9 [28], HOXC9 [29], HOXC10 [30], HOXD4 [31], HOXA11 [32] and HOXD9 [33]. These studies explored the prognostic value of HOX protein expression for determining OS or disease-free survival (DFS) and the correlation between the expression of HOX proteins and clinicopathological characteristics of patients with GC. HOX expression at the protein level was detected using immunohistochemical staining. All included studies divided HOX protein expression into high (positive) and low (negative) groups, but the cut-off value was slightly different among these studies. A detailed description of the characteristics of the included studies is provided in Table 1.

**Table 1:** Characteristics of the included studies

First author	Year	Country	HOX protein	Expression	Sample size (high/low)	Cut-off value	Survival	Survival analysis	HR availability	NOS score
Sha et al. [15]	2013	China	HOXB9	Downregulated	190 (86/104)	IS:4	OS	U, M	Text	7
Wang et al. [16]	2015	China	HOXD10	Downregulated	436 (242/194)	IS:4	OS	U	Kaplan-Meier curves	7
Peng et al. [17]	2018	China	HOXA5	Downregulated	81 (29/52)	IS:4	OS	U, M	Text	8
Wu et al. [18]	2019	China	HOXA5	Downregulated	124 (60/64)	median value	OS, DFS	U	Kaplan-Meier curves	7
Sentani et al. [19]	2012	Japan	HOXA10	Upregulated	749 (221/528)	percentage of stained cancer cells = 10%	OS	U, M	Text	7
Han et al. [22]	2013	China	HOXA13	Upregulated	132 (103/29)	IS:3	OS, DFS	U, M	Text	7
Lim et al. [20]	2013	Korea	HOXA10	Upregulated	57 (29/28)	compare to the staining smooth muscle	OS	U	Kaplan-Meier curves	7
Zhang et al. [24]	2013	China	HOXC6	Upregulated	161 (76/85)	IS:2	OS	U	Kaplan-Meier curves	8
Han et al. [21]	2015	China	HOXA10	Upregulated	264 (159/105)	IS:2	OS, DFS	U, M	Text	6
Tu et al. [25]	2015	China	HOXB7	Upregulated	96 (66/30)	IS:2	OS, DFS	U, M	Text	8
Yuan et al. [27]	2016	China	HOXA1	Upregulated	264 (144/120)	IS:3	OS, DFS	U	Text	6
He et al. [26]	2017	China	HOXB7	Upregulated	330 (195/135)	IS:4	OS	U	Kaplan-Meier curves	8
Ma et al. [28]	2017	China	HOXA9	Upregulated	128 (88/40)	IS:4	OS	U, M	Text	8
Han et al. [23]	2018	China	HOXA13	Upregulated	264 (186/78)	IS:3	OS, DFS	U, M	Text	6
Peng et al. [29]	2018	China	HOXC9	Upregulated	95 (68/27)	IS:4	OS	U, M	Text	7
Yao et al. [30]	2018	China	HOXC10	Upregulated	73 (38/35)	IS:4	OS	U, M	Text	7
Liu et al. [31]	2019	China	HOXD4	Upregulated	127 (68/59)	IS:7	OS	U, M	Text	7
Wang et al. [32]	2019	China	HOXA11	Upregulated	114 (58/56)	IOD/Area = 0.31	OS	U, M	Text	6
Zhu et al. [33]	2019	China	HOXD9	Upregulated	90 (55/35)	IS:3	OS	U	Kaplan-Meier curves	6

*OS* Overall survival, *DFS* Disease free survival, *U* Univariate analysis, *M* Multivariate analysis, *IS* Immunostaining score, *IOD* Integrated option density, *NOS* Newcastle-Ottawa Scale

### Correlation of HOX protein expression with the prognosis

**Fig. 2:**
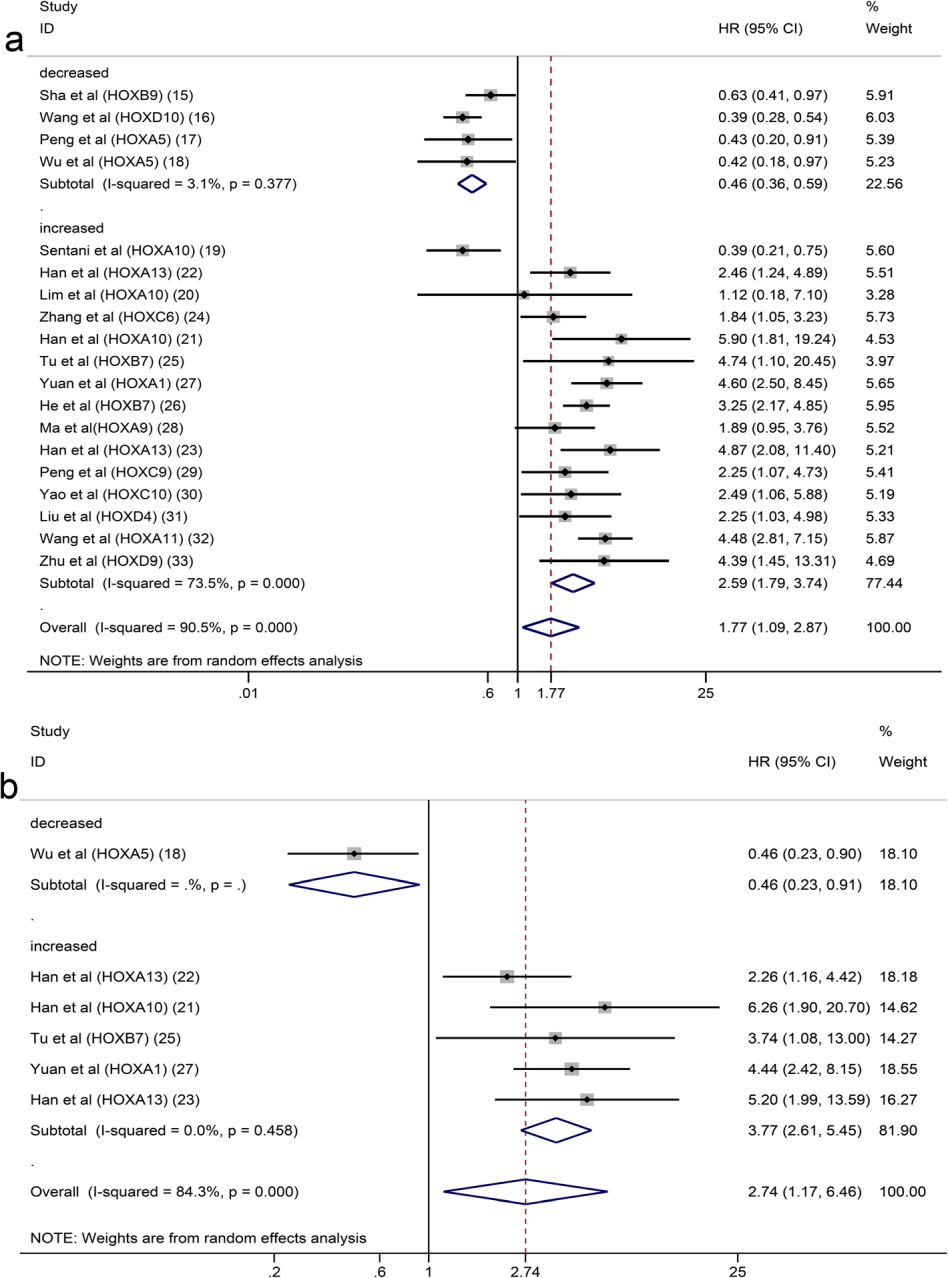
Subgroup analysis of OS (**a**) or DFS (**b**) by HOX protein expression in GC

**Fig. 3:**
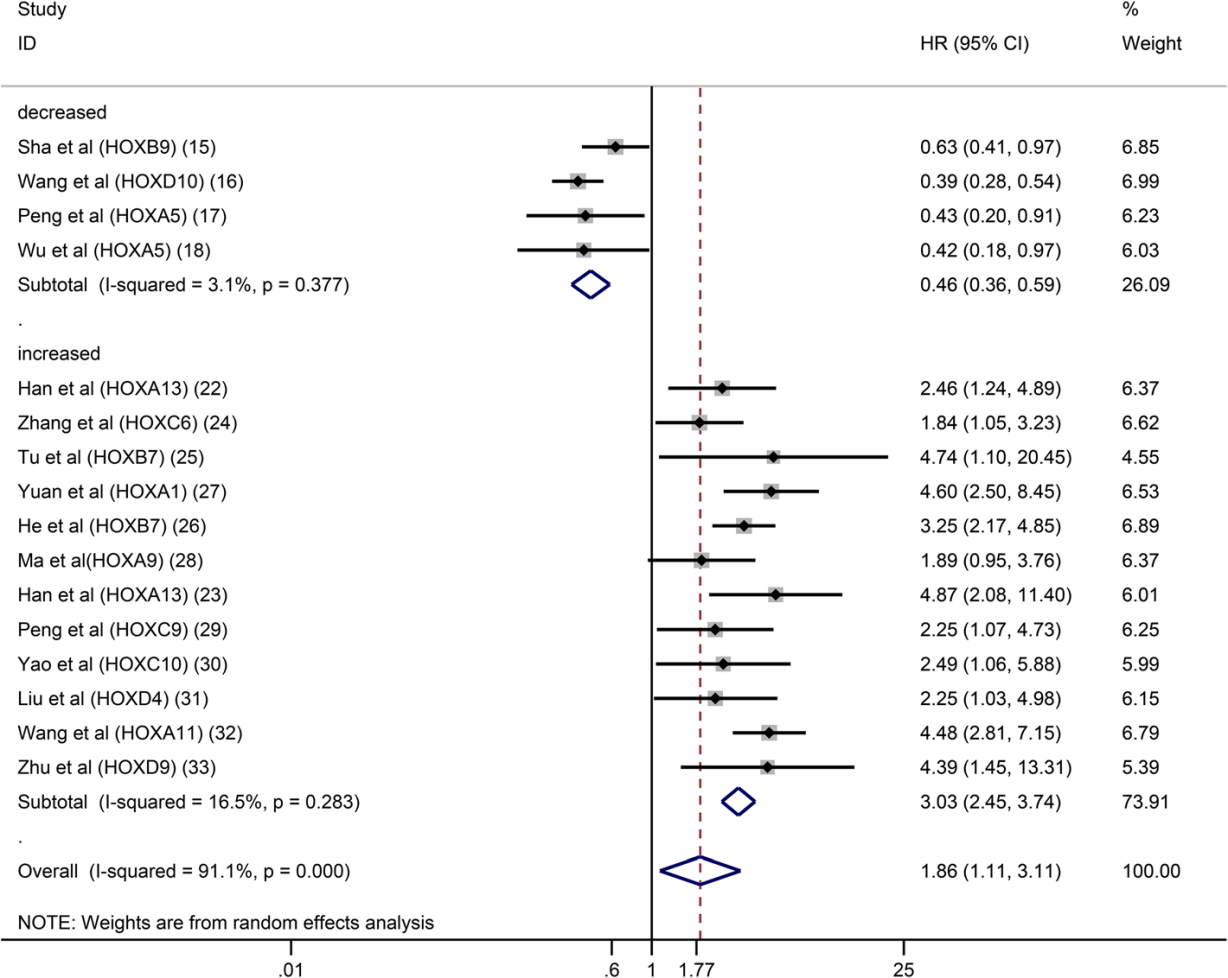
Subgroup analysis of OS by HOX protein expression in GC (excluded HOXA10)

This meta-analysis included a total of 19 articles containing 14 HOX proteins. HOXB9, HOXD10 and HOXA5 were expressed at low levels in GC and acted as tumour suppressors. In contrast, HOXA13, HOXC6, HOXB7, HOXA1, HOXC9, HOXC10, HOXD4, HOXA11 and HOXD9 were expressed at high levels and functioned as tumour promotors in patients with GC. In addition, HOXA10 expression was increased in GC, but its role in predicting the prognosis of GC was unclear. In a pooled analysis including all studies with data on the prognostic effects of HOX proteins in GC, considerable heterogeneity among pooled HRs for OS was observed. A subgroup analysis stratified by the expression level was performed, and the results revealed different trends between the downregulated subgroup and the upregulated subgroup. High expression of HOX proteins in the downregulated subgroup was associated with a good prognosis for patients with GC (pooled HR: 0.46, 95% CI: 0.36–0.59, I^2^ = 3.1%, *p* = 0.377), while the overexpression of HOX proteins in the upregulated subgroup was correlated with a poor OS (pooled HR: 2.59, 95% CI: 1.79–3.74, I^2^ = 73.5%, *p* = 0.000) **(**Fig. 2a**)**. The explanation for the high level of heterogeneity of the upregulated subgroup might be that HOXA10 had different prognostic values in the existing studies. The result of the analysis of the upregulated subgroup after excluding HOXA10 suggested that overexpressed HOX proteins significantly indicated a poor prognosis (pooled HR = 3.03, 95% CI: 2.45–3.74, I^2^ = 16.5%, *p* = 0.283) (Fig. 3). DFS was reported in 6 studies involving 5 HOX proteins. HOXA5 expression was associated with an increased DFS in patients with GC (pooled HR = 0.46, 95% CI: 0.23–0.91). In contrast, HOXA13, HOXA10, HOXB7 and HOXA1 expression was associated with a decreased DFS (pooled HR = 3.77, 95% CI: 2.61–5.45) (Fig. 2b).

### Correlation of HOX protein expression with clinicopathological characteristics

**Fig. 4:**
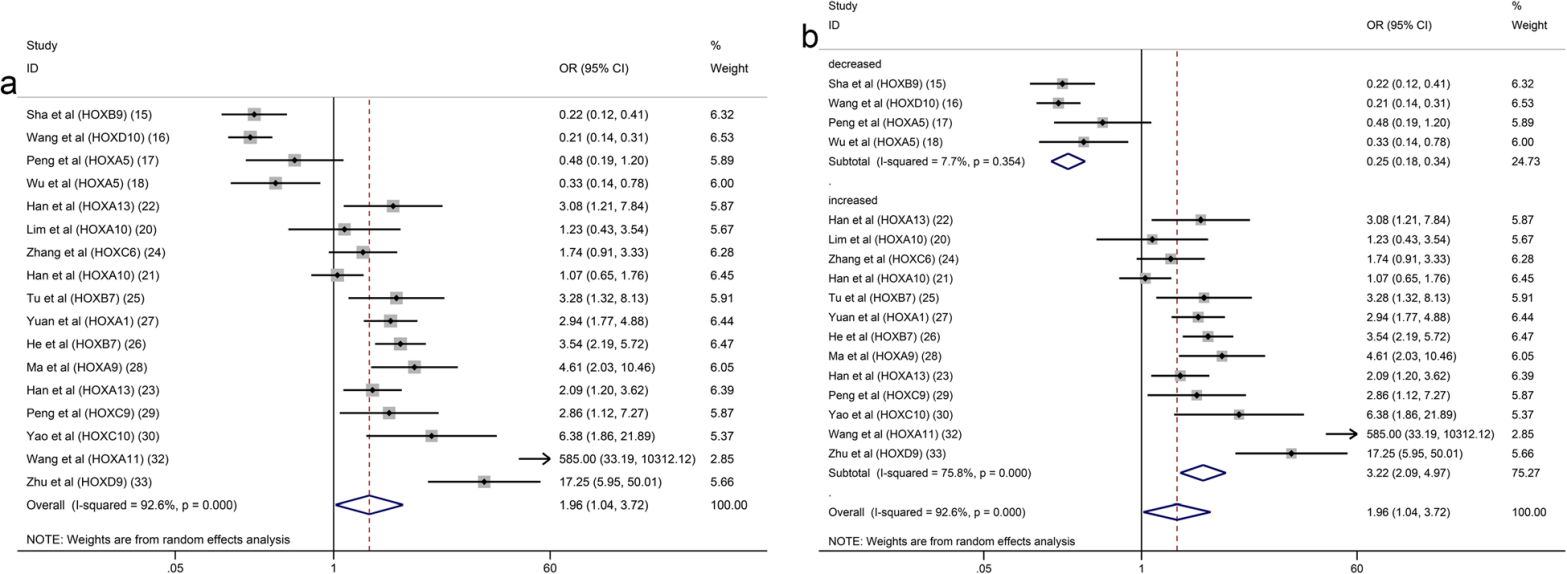
Forest plots of the pooled analysis for the association between HOX protein expression and TNM stage (**a**), TNM stage subgroup analysis (**b**)

**Fig. 5:**
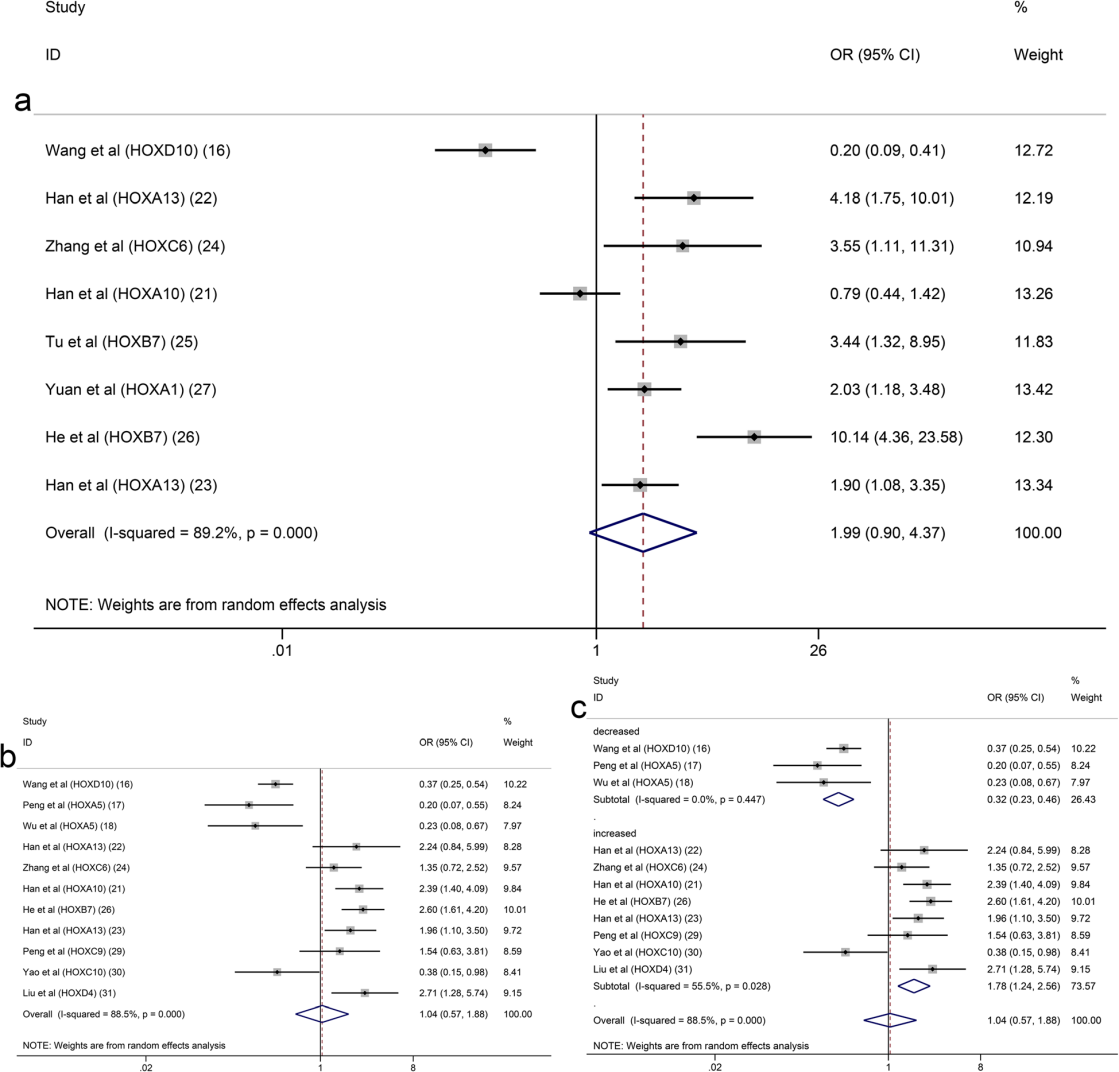
Forest plots of the pooled analysis for the association between HOX protein expression and T categories (**a**), tumour size (**b**), tumour size subgroup analysis (**c**)

**Fig. 6:**
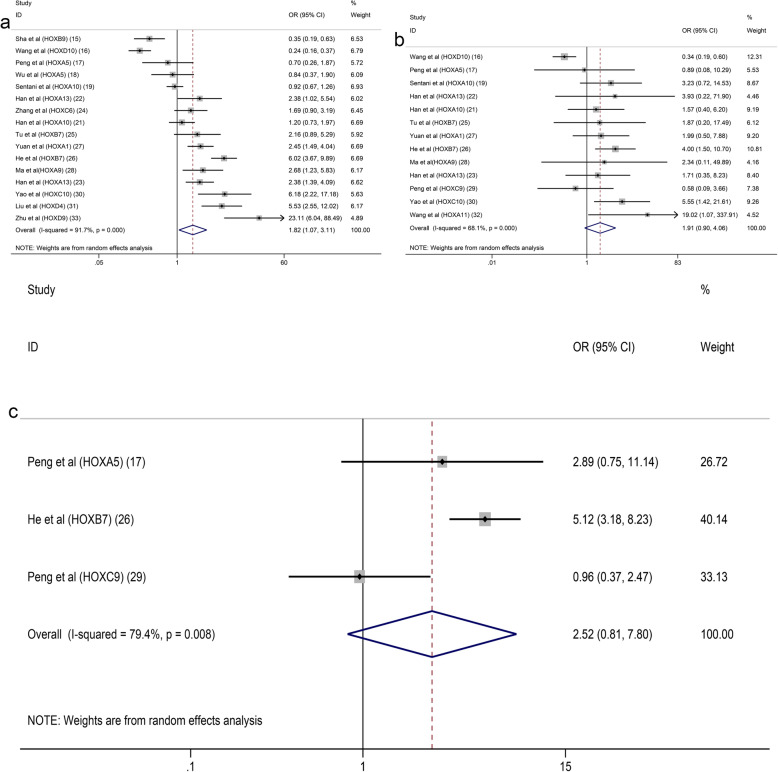
Forest plots of the pooled analysis for the association between HOX protein expression and lymph node metastasis (**a**), distant metastasis (**b**), vascular invasion (**c**)

**Fig. 7:**
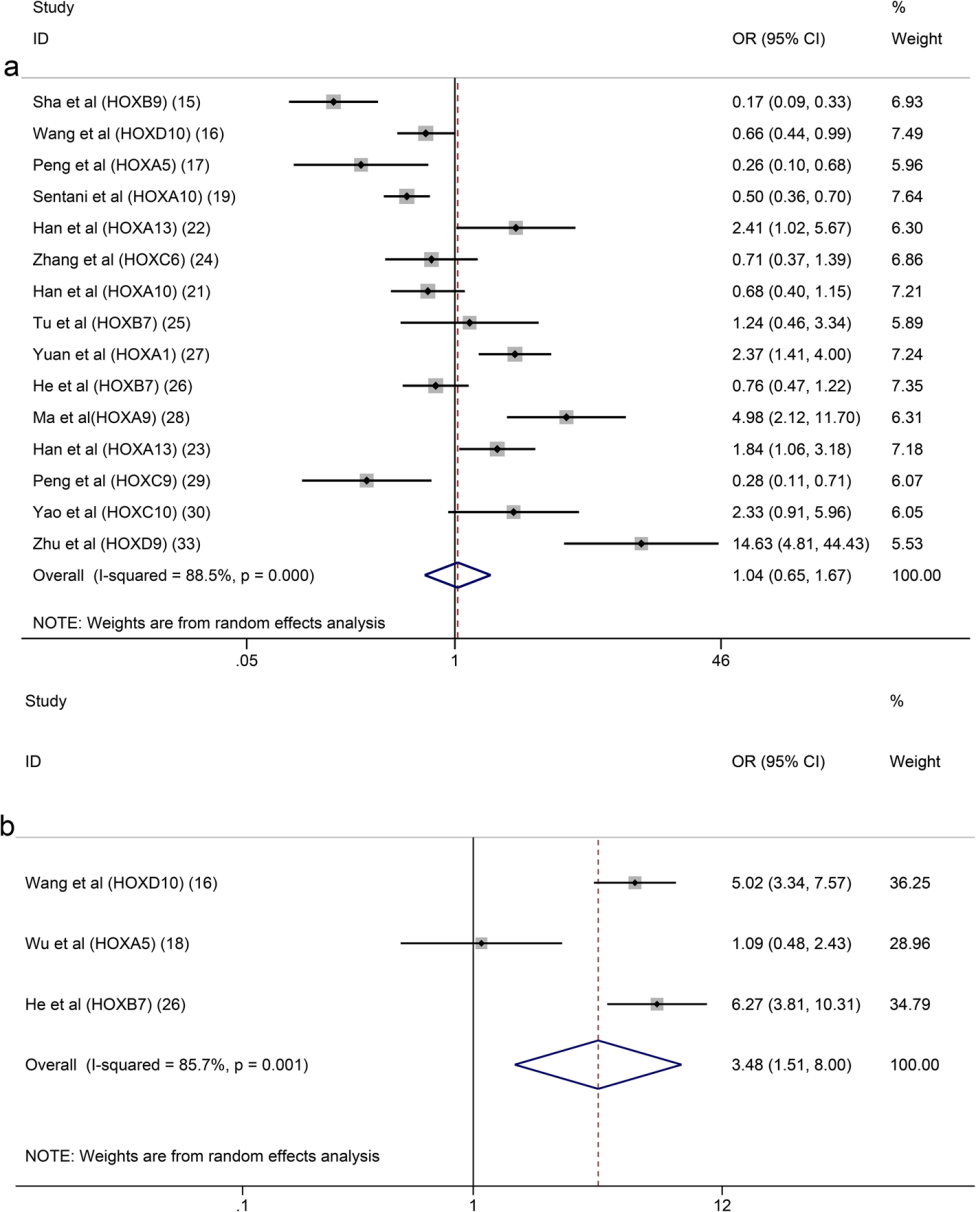
Forest plots of the pooled analysis for the association between HOX protein expression and histologic differentiation (**a**), Lauren classification (**b**)

**Fig. 8:**
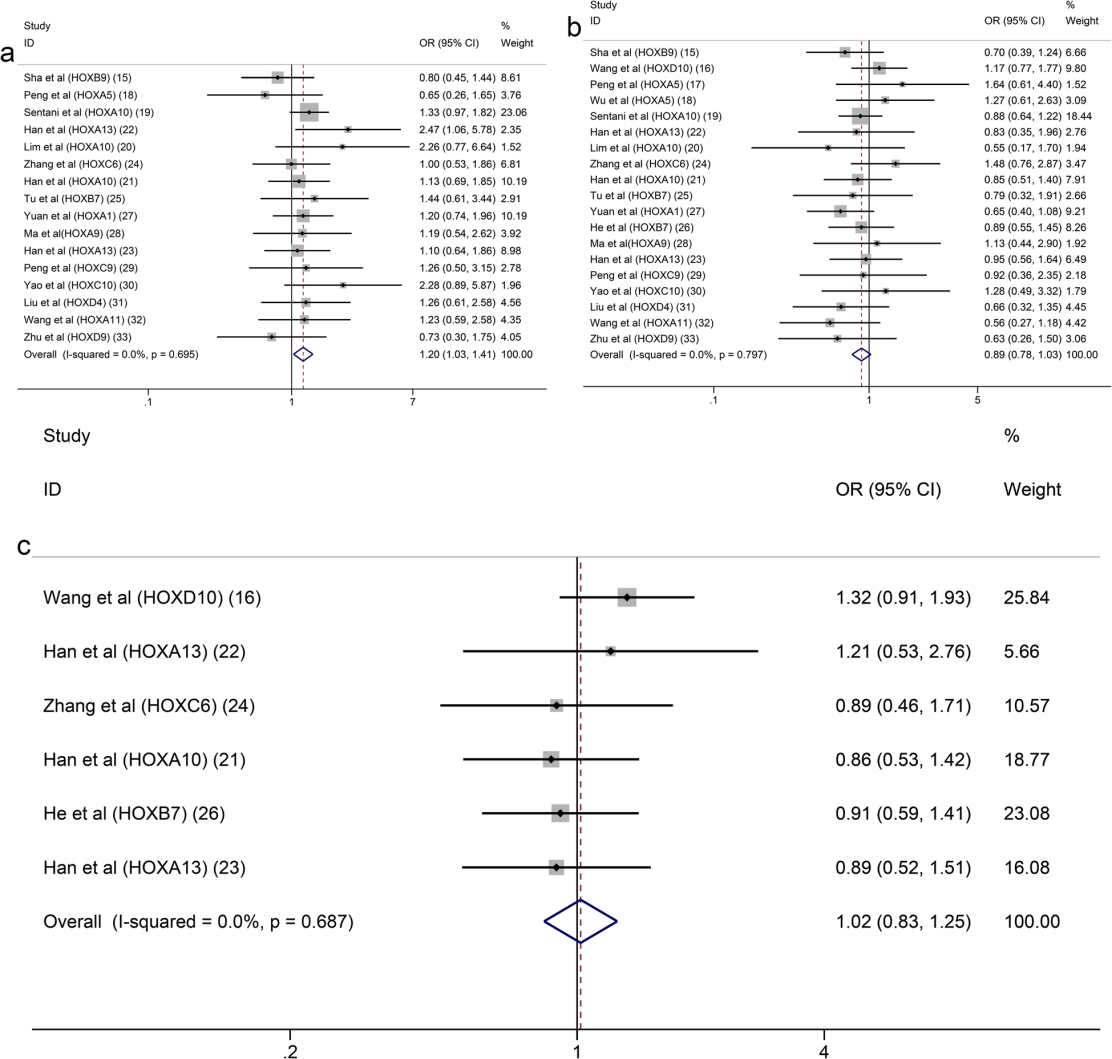
Forest plots of the pooled analysis for the association between HOX protein expression and age (**a**), sex (**b**), tumour location (**c**)

**Fig. 9:**
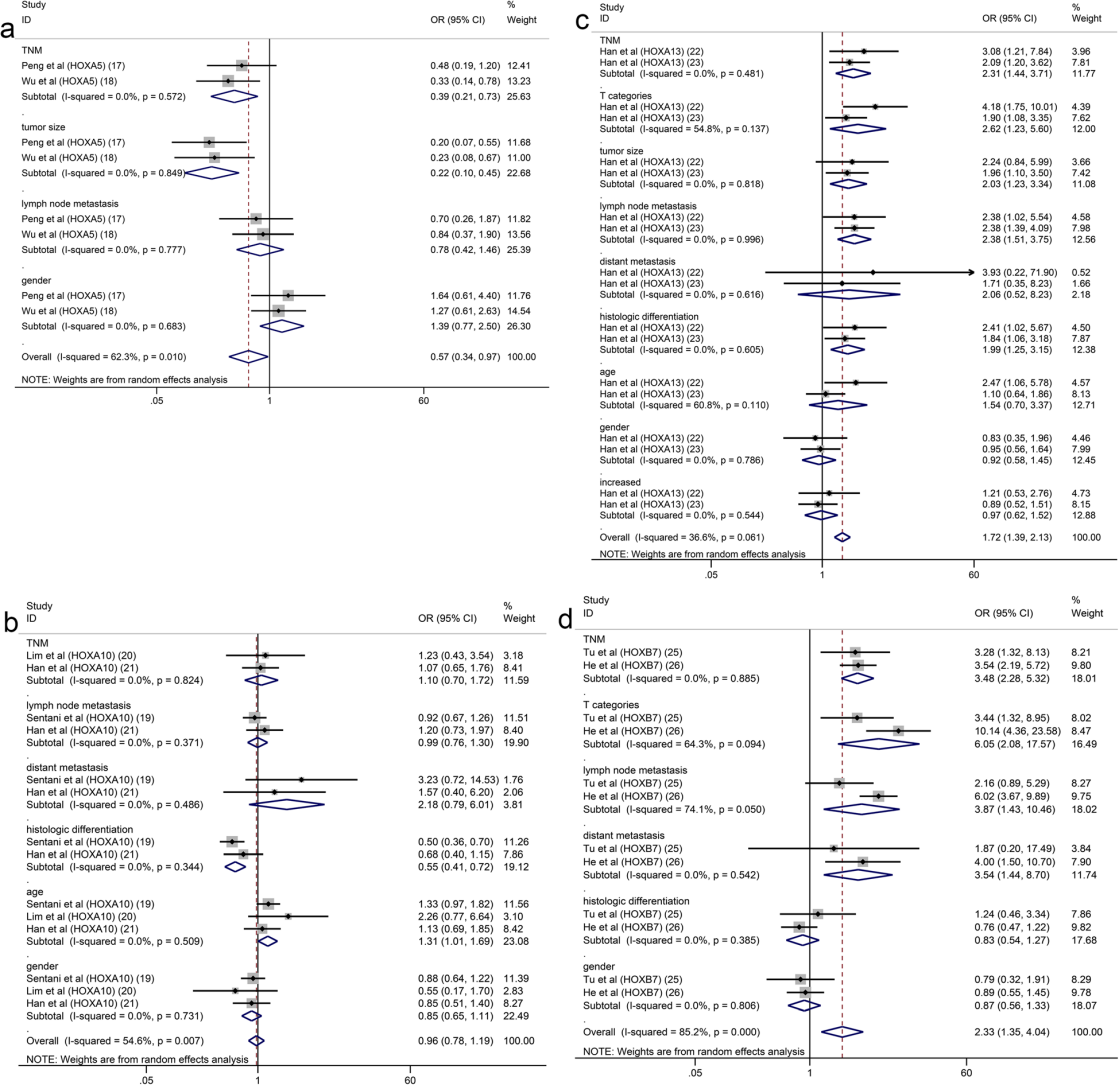
Forest plots of the pooled analysis for the association between HOX protein expression and clinicopathological characteristics: HOXA5 (**a**), HOXA10 (**b**), HOXA13 (**c**), HOXB7 (**d**)

Seventeen studies with 2899 patients were included to detect the relationship between HOX protein expression and tumour stage. As shown in Fig. 4a, increased expression of HOXB9 and HOXD10 was significantly correlated with an earlier TNM stage (HOXB9: OR = 0.22, 95% CI: 0.12–0.41, HOXD10: OR = 0.21, 95% CI:0.14–0.31), while increased expression of HOXA13, HOXB7, HOXA1, HOXA9, HOXC9, HOXC10, HOXA11 and HOXD9 was notably associated with an advanced TNM stage (I^2^ = 92.6%, *p* = 0.000). Due to the high level of heterogeneity, we performed a subgroup analysis based on the expression levels of HOX proteins. The heterogeneity of the upregulated group was decreased but still at a high level (I^2^ = 75.8%, *p* = 0.000) (Fig. 4b). A subsequent analysis showed that the studies of HOXA10 contributed a considerable amount of heterogeneity (data not shown). In addition, the difference of scoring systems for assessing expression levels of HOX proteins in the included studies was also one of the main sources of heterogeneity. The pooled analysis of the relationship between HOX proteins and the depth of tumour invasion showed that HOXD10 indicated a low T category (HOXD10: OR = 0.20, 95% CI: 0.09–0.41), while HOXA13, HOXC6, HOXB7 and HOXA1 were related to a high T category (HOXA13 (2013): OR = 4.18, 95% CI: 1.75–10.01; HOXA13 (2018): OR = 1.90, 95% CI: 1.08–3.35; HOXC6: OR = 3.55, 95% CI: 1.11–11.31; HOXB7 (2015): OR = 3.44, 95% CI: 1.32–8.95; HOXB7 (2017): OR = 10.14, 95% CI: 4.36–23.58; and HOXA1: OR = 2.03, 95% CI: 1.18–3.48) (Fig. 5a). We pooled 11 studies including 2087 patients and found that HOXD10, HOXA5 and HOXC10 were associated with a decreased tumour size (HOXD10: OR = 0.37, 95% CI: 0.25–0.54; HOXA5 (2018): OR = 0.20, 95% CI: 0.07–0.55; HOXA5 (2019): OR = 0.23, 95% CI: 0.08–0.67; and HOXC10: OR = 0.38, 95% CI: 0.15–0.98), while the overexpression of HOXA10, HOXB7 and HOXD4 was associated with an increased tumour size (HOXA10 (2015): OR = 2.39, 95% CI: 1.40–4.09; HOXB7 (2017): OR = 2.60, 95% CI: 1.61–4.20; and HOXD4: OR = 2.71, 95% CI: 1.28–5.74) (Fig. 5b). Similarly, the heterogeneity was significantly reduced by conducting a subgroup analysis according to expression levels of HOX proteins (Fig. 5c). Sixteen studies with 3509 patients reported that HOXB9 and HOXD10 were unfavourable factors for lymph node metastasis in patients with GC (HOXB9: OR = 0.35, 95% CI: 0.19–0.63 and HOXD10: OR = 0.24, 95% CI: 0.16–0.37), and overexpression of HOXA13, HOXA1, HOXA9, HOXC10, HOXD4 and HOXD9 was correlated with the presence of lymph node metastasis (HOXA13 (2013): OR = 2.38, 95% CI: 1.02–5.54; HOXA13 (2018): OR = 2.38, 95% CI: 1.39–4.09; HOXA1: OR = 2.45, 95% CI: 1.49–4.04; HOXA9: OR = 2.68, 95% CI: 1.23–5.83; HOXC10: OR = 6.18, 95% CI: 2.22–17.18; HOXD4: OR = 5.53, 95% CI: 2.55–12.02; and HOXD9: OR = 23.11, 95% CI: 6.04–88.49) (Fig. 6a). The results of the pooled analysis revealed that HOXD10 was not conducive to the distant metastasis of GC (HOXD10: OR = 0.34, 95% CI: 0.19–0.60), but that HOXC10 and HOXA11 promoted distant metastasis of GC (HOXC10: OR = 5.55, 95% CI: 1.42–21.61 and HOXA11: OR = 19.02, 95% CI: 1.07–337.91) (Fig. 6b). In addition, the upregulation of HOXB7 promoted vascular invasion in patients with GC (HOXB7 (2017): OR = 5.12, 95% CI: 3.18–8.23) (Fig. 6c). Moreover, HOXB9, HOXD10, HOXA5 and HOXC9 were factors contributing to good or moderate histological differentiation (HOXB9: OR = 0.17, 95% CI: 0.09–0.33, HOXD10: OR = 0.66, 95% CI: 0.44–0.99, HOXA5 (2018): OR = 0.26, 95% CI: 0.10–0.68; and HOXC9: OR = 0.28, 95% CI: 0.11–0.71), and overexpression of HOXA13, HOXA1, HOXA9 and HOXD9 was related to poorly differentiated status of GC (HOXA13 (2013): OR = 2.41, 95% CI: 1.02–5.67; HOXA13 (2018): OR = 1.84, 95% CI: 1.06–3.18; HOXA1: OR = 2.37, 95% CI: 1.41–4.00; HOXA9: OR = 4.98, 95% CI: 2.12 11.70; and HOXD9: OR = 14.63, 95% CI: 4.81–44.43) (Fig. 7a). Additionally, HOXD10 and HOXB7 was correlated with the intestinal phenotype of GC (HOXD10: OR = 5.02, 95% CI: 3.34–7.57 and HOXB7 (2017): OR = 6.27, 95% CI: 3.81–10.31) (Fig. 7b). None of the HOX proteins included in the pooled analysis exhibited significant associations with age (Fig. 8a), sex (Fig. 8b) or tumour location (Fig. 8c). Additionally, the relationships between HOXA5, HOXA10, HOXA13 and HOXB7 expression and clinicopathological characteristics were all explored in several studies. As shown in Fig. 9, HOXA5 expression predicted a smaller tumour size (OR = 0.22, 95% CI: 0.10–0.45) (Fig. 9a), but there is no correlation between HOXA10 expression and clinicopathological features (Fig. 9b). The overexpression of both HOXA13 (Fig. 9c) and HOXB7 (Fig. 9d) was significantly associated with an advanced tumour stage (HOXA13: OR = 2.31, 95% CI: 1.44–3.71 and HOXB7: OR = 3.48, 95% CI: 2.28–5.32) and a high T category (HOXA13: OR = 2.62, 95% CI: 1.23–5.60 and HOXB7: OR = 6.05, 95% CI: 2.08–17.57), and HOXA13 was also related to lymph node metastasis (OR = 2.38, 95% CI: 1.51–3.75) and poor differentiation status (OR = 1.99, 95% CI: 1.25–3.15).

### Sensitivity analysis

**Fig. 10:**
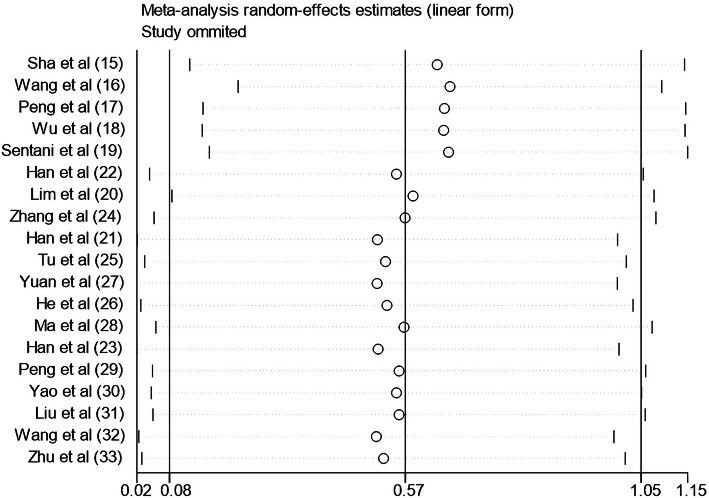
Sensitivity analysis of the included studies on OS

A sensitivity analysis was performed to verify the robustness of our results. As shown in Fig. 10, the pooled HR was not significantly altered when each study was removed, which confirmed the reliability of overall results for the OS of patients with GC.

### Publication bias

**Fig. 11:**
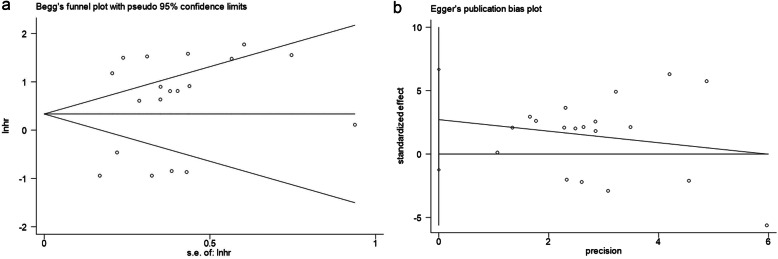
Tests for publication bias of OS: Begg’s test (**a**), Egger’s test (**b**)

Begg’s test and Egger’s test were performed to evaluate publication bias. The results did not reveal substantial publication bias (Fig. 11: Begg’s test: *p* = 0.576, Egger’s test: *p* = 0.166).

### Mechanisms by which HOX proteins regulate GC

In Table 2 and supplementary Fig. 1, we summarize the molecular mechanisms by which HOX proteins included in this study modulate carcinogenesis and development of GC [15–57]. HOXB9 inhibits GC progression via AKT and NF-κB pathways [34]. HOXD10 suppresses the migration and invasion of GC cells through insulin-like growth factor binding protein-3 (IGFBP3) and RhoC-AKT pathway [36, 39]. HOXA5 suppresses GC progression by inhibiting the G1/S transition during the cell cycle [17]. HOXA13 promotes GC development via TGF-β, ERK1/2, MDM2-p53- MRP1 pathways, and Wnt/β-catenin signalling [23, 44–46]. HOXC6 enhances invasive and metastatic abilities of GC cells by upregulating the expression of MMP9 via activating ERK pathway [48]. HOXA1 increases the proliferation of GC cells by upregulating cyclin D1 expression [27]. HOXB7 mediates GC cell malignancy by activating AKT/MAPK signalling, Src-FAK pathway, PIK3R3/AKT pathway, and epithelial mesenchymal transition (EMT) [26, 49, 50]. The miR-182/HOXA9 axis is implicated in RUNX3-mediated GC development [51]. In addition, HOXC9 contributes to GC progression by inducing EMT, MMP2 expression, and stem cell-like properties [29]. HOXC10 activates ATM/NF-kB pathway and MAPK signalling, functioning as an oncogene in GC [30, 54]. HOXD4 increases the proliferation and invasion of GC cells by upregulating c-Myc and cyclin D1 [31]. HOXD9 activates RUFY3, increasing the proliferation, migration and invasion of GC cells [33]. However, the effects of HOXA10 and HOXA11 on tumorigenesis and development of GC are controversial.

**Table 2:** Action mechanisms of HOX proteins in gastric cancer

HOX proteins	Expression	Upstream	Downstream	Pathways	Reference
HOXB9	Downregulated	NA	NA	↓cells proliferation, migration and invasion; ↑MET; AKT and NF-κB pathway	[15, 34, 35]
HOXD10	Downregulated	miR-10b, miR-92b-3p	IGFBP3	↓cells proliferation, migration and invasion; AKT pathway; RhoC pathway	[16, 36–39]
HOXA5	Downregulated	miR-196a	NA	↓cells G1-S transition, proliferation and colony formation; ↓angiogenesis	[17, 18]
HOXA10	Upregulated	NA	miR-196b-5p, BCL2	↑cells viability, proliferation, colony information, migration and invasion ↓apoptosis; ↑tumor metastasis; JAK1/STAT3 signaling; HOXA10/miR-196b-5p axis; ↓cells growth, motility and invasive activity;	[19–21, 40–42]
HOXA13	Upregulated	lncRNA HOTTIP	DHRS2, cadherin17	↑cells proliferation, migration and invasion; ↑EMT; TGF-β pathway, ERK1/2 pathway, Wnt/β-catenin pathway, MDM2-p53-MRP1 pathway; chemotherapy resistance to 5-FU	[22, 23, 43–46]
HOXC6	Upregulated	lncRNA HOTAIR	NA	↑cells proliferation, colony formation, migration and invasion; ERK signaling;	[24, 47, 48]
HOXB7	Upregulated	NA	NA	↑cells G1-S transition, proliferation, migration and invasion; ↑EMT; ↓apoptosis; AKT/MAPK pathway; Src-FAK pathway; PIK3R3/ AKT pathway	[25, 26, 49, 50]
HOXA1	Upregulated	NA	NA	↑cells proliferation, invasion and migration; ↑cyclin D1	[27]
HOXA9	Upregulated	miR-182	NA	↑cells proliferation, migration and invasion; ↑tumor progression	[28, 51]
HOXC9	Upregulated	miR-26a	NA	↑EMT and stem cell-like phenotypic acquisition; ↑tumor metastasis	[29]
HOXC10	Upregulated	miR-136	CST1	↑cells migration and invasion; ↑tumor growth and peritoneal metastasis; ATM/NF-kB pathway; MAPK signaling	[30, 52–55]
HOXD4	Upregulated	NA	NA	↑cells proliferation, migration and invasion; ↑c-Myc and cyclinD1	[31]
HOXA11	Controversial	STAT3	STAT3	Wnt pathway	[32, 56, 57]
HOXD9	Upregulated	NA	RUFY3	↑cells proliferation, invasion and migration; ↑tumorigenesis and metastasis	[33]

↓: inhibit; ↑: promote; *NA* Not available, *AKT* Protein kinase B, *ATM* Ataxia telangiectasia mutated, *BCL2* B cell lymphoma-2, *CST1* Cystatin 1, *DHRS2* Dehydrogenase/reductase 2, *ERK* Extracellular regulated protein kinases, *FAK* Focal adhesion kinase, *IGFBP3* Insulin-like growth factor binding protein-3, *JAK1* Janus kinase 1, *MAPK* Mitogen-activated protein kinase, *MDM2* Murine double minute 2, *MET* Mesenchymal epithelial transition, *MRP1* Multidrug resistance-associated protein 1, *PIK3R3* Phosphoinositide-3-kinase, regulatory subunit 3, *RhoC* Ras superfamily of GTP-binding protein, *Src* Steroid receptor coactivator, *RUFY3* RUN and FYVE domain containing 3, *5-FU* 5-fluorouracil

## Discussion

GC is a main cause of cancer-related mortality. Currently, radical gastrectomy combined with adjuvant chemotherapy is recognized as the most effective treatment for GC. Nevertheless, many patients with GC are usually diagnosed in an advanced stage, missing the opportunity for radical surgical resection. Based on the current situation, it is important to identify factors which is helpful to improving prediction accuracy and promoting curative effect of GC. Most of the HOX genes are generally activated and expressed during embryogenesis, and many of these proteins are aberrantly expressed during tumorigenesis. According to the literatures, HOX proteins are related to the prognosis and clinicopathological features of GC, but the results are controversial. We conducted this study to further clarify the effects of HOX proteins on the prognosis and clinicopathological characteristics of GC and describe the molecular mechanisms by which HOX proteins regulate tumorigenesis and development of GC.

The present systematic review and meta-analysis enrolled 19 eligible studies containing 3775 patients. In the pooled analysis of the effects of HOX proteins on the GC prognosis, HOXB9, HOXD10 and HOXA5 were correlated with a good prognosis in patients with GC, while HOXA13, HOXC6, HOXB7, HOXA1, HOXC9, HOXC10, HOXD4, HOXA1 and HOXD9 were related to a poor prognosis. However, Kato et al. identified positive HOXB9 expression in GC as a marker of a poor prognosis. Unfortunately, the study by Kato was not included in this meta-analysis due to the lack of an analysis of HOXB9 expression in paired noncancerous mucosae [58].

The relationship between HOX proteins and clinicopathological features of GC were also analysed in this study. The results revealed correlations between the expression of HOX proteins and TNM stage, T category, tumour size, lymph node metastasis, distant metastasis, vascular invasion, histological differentiation, and Lauren classification in GC. Based on the results of the meta-analysis described above, we speculated that HOX proteins might predict the prognosis of patients with GC, which was also confirmed in each included original study. Therefore, we inferred that combined detection of the expression of various HOX proteins might provide a novel perspective for predicting the prognosis of patients with GC.

Currently, some clinicopathological parameters such as age, sex, tumour stage, depth of invasion, lymph node metastasis, distant metastasis, and resection margins, have been proven to be prognostic indicators of GC [59, 60]. At the same time, several molecules are under investigation as predictors of survival, such as gene mutations, DNA methylation, RNAs, and proteins [61]. Regrettably, many studies have only explored the individual relationship between clinicopathological characteristics or molecular markers and the prognosis of patients with GC, although a few studies have established prognostic models [62]. Bria et al. combined clinicopathological parameters (sex, age, Lauren classification, stage, margins, grade, site, size, and resected nodes) with molecular markers (HER2, FHIT, and APC) to construct a risk stratification of GC, establishing a scientific model to determine its prognosis. In addition, the authors conducted a large prospective validation with a larger sample size to eliminate all sources of bias in the retrospective study [63]. GC is highly heterogeneous, and even similar clinicopathological features result in different outcomes, suggesting that a more reasonable classification system is needed for predicting the prognosis and therapeutic effect of GC. A novel classification system with four molecular subtypes was developed by The Cancer Genome Atlas (TCGA) [64]. Besides, Sohn et al. developed a scoring system (TCGA risk score) based on TCGA to predict prognosis and adjuvant chemotherapy outcomes in patients with GC, which was validated as an independent prognostic factor for GC in multivariate Cox regression analyses [65]. Analogously, Lin et al. established a novel prognosis scoring system based on TCGA and Gene Expression Omnibus to predict the prognosis of GC, which comprised signatures of tumour protein-coding genes (P), tumour noncoding genes (N) and immune/stroma cells in the tumour microenvironment (M) (PMC score). Furthermore, the combination of PNM scores with American Joint Committeeon Cancer (AJCC) staging significantly increased its predictive value [66]. In addition, Tahara et al. investigated the prognosis and clinicopathological characteristics of GC by combining genetic and epigenetic abnormalities. The CpG island methylator phenotype (CIMP) and TP53 hot spot mutation status (R175, G245, R248, R273, and R282) were sufficient to predict the prognosis and clinicopathological features of GC. Among these features, patients with the CIMP^−^TP53 hot spot^+^ subtype presented the worst overall survival [67]. Moreover, Ooi et al. selected three oncogenic pathways (NF-κB, Wnt/β-catenin, and proliferation/stem cells) by analysing a GC pathway heatmap and combined them to predict its prognosis, which was validated in vitro [68].

The development of GC is determined by both genes and environmental factors, which has been confirmed in mouse models. Microbial infections, particularly *Helicobacter pylori* (*H. pylori*) and Epstein-Barr virus, are important environmental factors and have been confirmed as prognostic factors for GC [69, 70]. Although *H. pylori* infection is the strongest risk factor for GC, very few *H. pylori*-infected populations develop GC. This outcome is attributed to the duration of infection, strain type and host genetic signatures [71]. The crucial effects of genetic factors on GC development have been revealed using progress in genetic technology, including the construction of genetically engineered mice via recombinant DNA technology to achieve molecular overexpression or deficiency, as well as gene mutations, clarifying the pathogenesis of GC and the interactions between various factors. For example, INS-GAS transgenic mice on the FVB genetic background that overexpress gastrin develop intramucosal carcinomas with submucosal and intravascular invasion in less than 1 year when infected by *Helicobacter felis* (*H. felis*) or *H. pylori*, with males showing a higher prevalence than females, indicating sex differences in GC tumorigenesis [72, 73]. However, INS-GAS mice on a C57BL/6 background infected with *H. felis* do not progress to GC [74]. Surprisingly, gastrin knockout mice (GAS−/− mice) are also confirmed to be susceptible to GC and exhibit antral GC, in contrast to INS-GAS mice, which develop corpus cancers [75]. Moreover, GAS−/− mice are more susceptible to antral cancer induced by MNU, a gastric carcinogen used in mouse models, compared to WT mice on the same genetic background [76].

Taken together, these studies reveal important roles of genetic signatures in the development of GC, and the external factor such as infection is also indispensable. Thus, the establishment of a comprehensive and detailed scoring system containing the most basic clinicopathological parameters, molecular markers, gene expression profiles, microbial infections, etc., might be more accurate in predicting the prognosis of patients with GC than a single factor. Our manuscript analysing the effects of HOX proteins in GC development aimed to predict the prognosis and provide therapeutic targets for GC. The results of this meta-analysis recommend the inclusion of HOX proteins in the model predicting the prognosis of GC.

Several limitations of this systematic review and meta-analysis should be noted. First, several HRs and their 95% CIs for OS were extracted from the survival curves, which might affect the reliability of the results. Second, the sample size of each study was not large enough, which might affect the accuracy of the results. Third, IHC methodologies including the primary antibody used, antibody dilutions, and the scoring system applied, differed, which might partially contribute to the heterogeneity. Finally, all patients included in our study were Asians, which might restrict the applicability of our results to other races.

## Conclusions

This systematic review and meta-analysis firstly generalized and evaluated the significance of HOX proteins in modulating the prognosis and clinicopathological characteristics of GC. We also summarized the molecular mechanisms by which HOX proteins regulate tumorigenesis and development of GC. Based on these findings, HOX proteins might serve as biomarkers and therapeutic targets for GC. Considering the limitations of this study, further large-scale prospective and high-quality studies are required to confirm the potential values of HOX proteins in GC.

## Supplementary information

**Additional file 1: Figure S1.** Molecular mechanisms how HOX proteins regulate tumorigenesis and development of GC.↑: promote; ⊥: inhibit; AKT: protein kinase B; ATM: ataxia telangiectasia mutated; BCL2: B cell lymphoma-2; CDH17: cadherin 17; CST1: cystatin SN; DHRS2: dehydrogenase/reductase 2; EGF: epidermal growth factor; ERK: extracellular regulated protein kinases; FAK: focal adhesion kinase; IGFBP3: insulin-like growth factor binding protein-3; JAK1: janus kinase 1; MAPK: mitogen-activated protein kinase; MDM2: murine double minute 2; MET: mesenchymal epithelial transition; MMP2: matrix metalloproteinase 2; MMP9: matrix metalloproteinase 9; MMP14: matrix metalloproteinase 14; MRP1: multidrug resistance-associated protein 1; NF-κB: nuclear factor-kappa B; NKD1: naked cuticle homolog 1; PIK3R3: phosphoinositide-3-kinase, regulatory subunit 3; RhoC: ras superfamily of GTP-binding protein; RUFY3: RUN and FYVE domain containing 3; RUNX3: runt-related transcription factor 3; Src: steroid receptor coactivator; STAT3: signal transducers and activators of transcription 3; TFF1: trefoil factor 1; TGF-β: transforming growth factor-β; TNF-α: tumour necrosis factor-α; uPA: urokinase-type plasminogen activator; uPAR: urokinase-type plasminogen activator receptor.

## Data Availability

All data generated or analysed in this study are included in this published article.

## Abbreviations

AJCC: American Joint Committeeon Cancer
AKT: Protein kinase B
ATM: Ataxia telangiectasia mutated
CI: Confidence interval
CIMP: CpG island methylator phenotype
DFS: Disease-free survival
EMT: Epithelial mesenchymal transition
ERK: Extracellular regulated protein kinases
FAK: Focal adhesion kinase
GC: Gastric cancer
*H. felis*: *Helicobacter felis*
HOX: Homeobox
HR: Hazard ratio
IGFBP3: Insulin-like growth factor binding protein-3
IHC: Immunohistochemical
MAPK: Mitogen-activated protein kinase
MDM2: Murine double minute 2
MRP1: Multidrug resistance-associated protein 1
NF-κB: Nuclear factor-kappa B
NOS: Newcastle-Ottawa Quality Assessment Scale
OR: Odds ratio
OS: Overall survival
PIK3R3: Phosphoinositide-3-kinase, regulatory subunit 3
RhoC: Ras superfamily of GTP-binding protein
RUFY3: RUN and FYVE domain containing 3
TGF-β: Transforming growth factor-β
