# Supplementary material for: Homeobox proteins are potential biomarkers and therapeutic targets in gastric cancer: a systematic review and meta-analysis
Source: BMC Cancer. 2020 Sep 9;20:866. doi: 10.1186/s12885-020-07346-7 (PMC7487678; doi:10.1186/s12885-020-07346-7)
Supplement: Supplementary file 1 — Additional file 1: Figure S1. Molecular mechanisms how HOX proteins regulate tumorigenesis and development of GC.↑: promote; ⊥: inhibit; AKT: protein kinase B; ATM: ataxia telangiectasia mutated; BCL2: B cell lymphoma-2; CDH17: cadherin 17; CST1: cystatin SN; DHRS2: dehydrogenase/reductase 2; EGF: epidermal growth factor; ERK: extracellular regulated protein kinases; FAK: focal adhesion kinase; IGFBP3: insulin-like growth factor binding protein-3; JAK1: janus kinase 1; MAPK: mitogen-activated protein kinase; MDM2: murine double minute 2; MET: mesenchymal epithelial transition; MMP2: matrix metalloproteinase 2; MMP9: matrix metalloproteinase 9; MMP14: matrix metalloproteinase 14; MRP1: multidrug resistance-associated protein 1; NF-κB: nuclear factor-kappa B; NKD1: naked cuticle homolog 1; PIK3R3: phosphoinositide-3-kinase, regulatory subunit 3; RhoC: ras superfamily of GTP-binding protein; RUFY3: RUN and FYVE domain containing 3; RUNX3: runt-related transcription factor 3; Src: steroid receptor coactivator; STAT3: signal transducers and activators of transcription 3; TFF1: trefoil factor 1; TGF-β: transforming growth factor-β; TNF-α: tumour necrosis factor-α; uPA: urokinase-type plasminogen activator; uPAR: urokinase-type plasminogen activator receptor. [file 12885_2020_7346_MOESM1_ESM.ppt]

## Slide 1
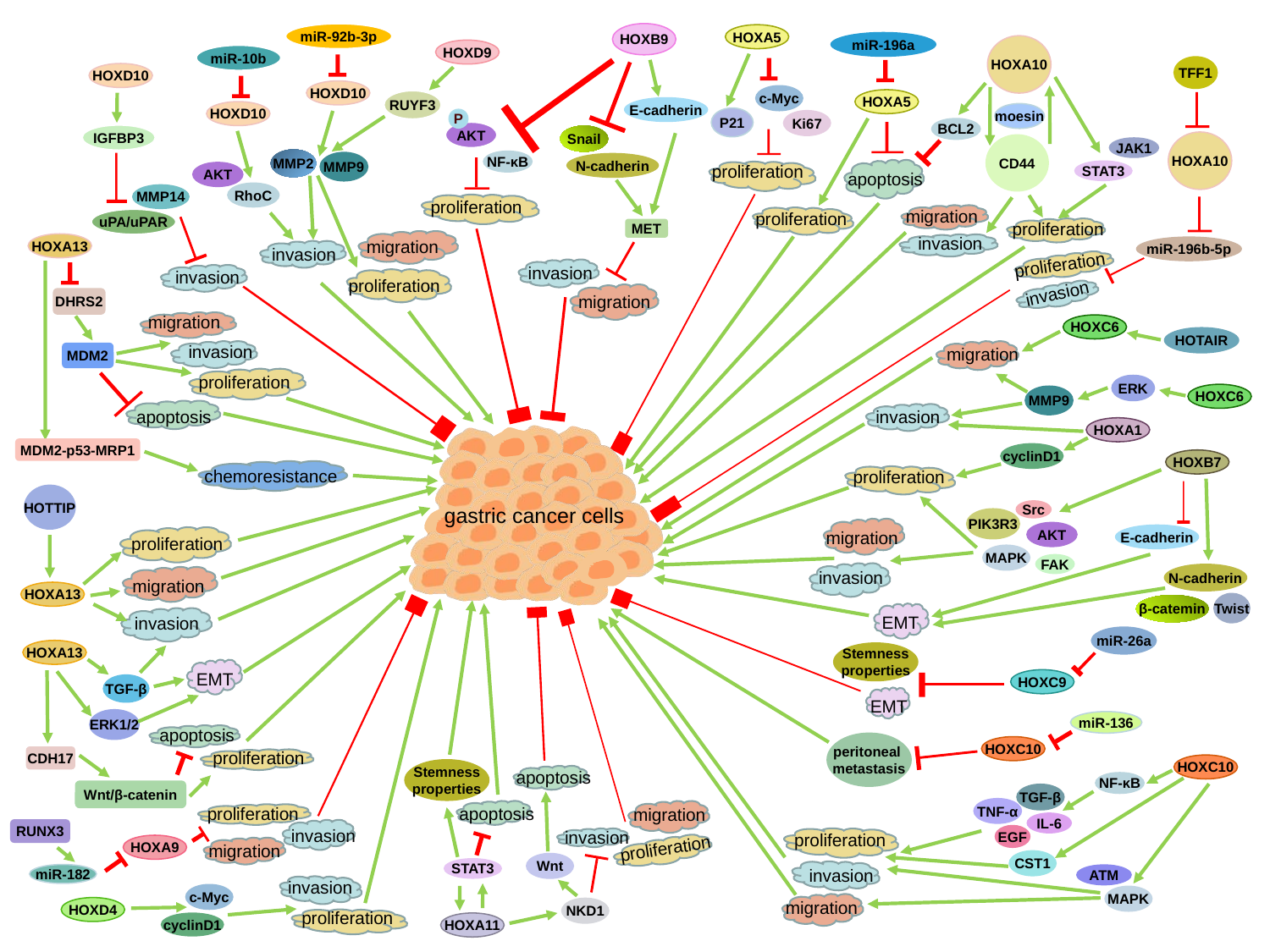

HOXB9
miR-92b-3p
HOXA5
miR-196a
HOXA10
HOXD9
miR-10b
TFF1
HOXD10
HOXD10
c-Myc
HOXA5
RUYF3
E-cadherin
HOXD10
moesin
P21
P
Ki67
BCL2
AKT
IGFBP3
Snail
HOXA10
CD44
JAK1
MMP2
NF-κB
MMP9
N-cadherin
proliferation
STAT3
AKT
apoptosis
RhoC
MMP14
proliferation
migration
proliferation
uPA/uPAR
proliferation
MET
invasion
migration
HOXA13
miR-196b-5p
invasion
proliferation
invasion
invasion
proliferation
invasion
migration
DHRS2
migration
HOXC6
HOTAIR
invasion
migration
MDM2
proliferation
ERK
HOXC6
MMP9
apoptosis
invasion
HOXA1
MDM2-p53-MRP1
cyclinD1
HOXB7
chemoresistance
proliferation
HOTTIP
gastric cancer cells
Src
PIK3R3
migration
AKT
E-cadherin
proliferation
MAPK
FAK
invasion
N-cadherin
migration
HOXA13
Twist
β-catemin
EMT
invasion
miR-26a
HOXA13
Stemness
properties
EMT
HOXC9
TGF-β
EMT
ERK1/2
miR-136
apoptosis
peritoneal
metastasis
HOXC10
proliferation
CDH17
HOXC10
Stemness
properties
apoptosis
NF-κB
Wnt/β-catenin
TGF-β
proliferation
apoptosis
migration
TNF-α
IL-6
invasion
RUNX3
invasion
proliferation
EGF
proliferation
migration
HOXA9
CST1
Wnt
STAT3
invasion
ATM
miR-182
invasion
c-Myc
MAPK
migration
HOXD4
NKD1
proliferation
cyclinD1
HOXA11
